# Growth and Characterization of Lead-free Piezoelectric Single Crystals

**DOI:** 10.3390/ma8115436

**Published:** 2015-11-24

**Authors:** Philippe Veber, Feres Benabdallah, Hairui Liu, Gabriel Buse, Michael Josse, Mario Maglione

**Affiliations:** 1 Institut de Chimie de la Matière Condensée de Bordeaux (ICMCB), Unité Propre de Recherche (UPR) 9048, Centre National de la Recherche Scientifique (CNRS), Pessac F-33600, France; benabdallah@icmcb-bordeaux.cnrs.fr (F.B.); hairui.liu@icmcb.cnrs.fr (H.L.); gabriel.buse@icmcb.cnrs.fr (G.B.); michael.josse@icmcb.cnrs.fr (M.J.); mario.maglione@icmcb.cnrs.fr (M.M.); 2 ICMCB, UPR 9048, Université de Bordeaux, Pessac F-33600, France

**Keywords:** crystal growth, lead-free materials, piezoelectrics

## Abstract

Lead-free piezoelectric materials attract more and more attention owing to the environmental toxicity of lead-containing materials. In this work, we review our first attempts of single crystal grown by the top-seeded solution growth method of BaTiO_3_ substituted with zirconium and calcium (BCTZ) and (K_0.5_Na_0.5_)NbO_3_ substituted with lithium, tantalum, and antimony (KNLSTN). The growth methodology is optimized in order to reach the best compositions where enhanced properties are expected. Chemical analysis and electrical characterizations are presented for both kinds of crystals. The compositionally-dependent electrical performance is investigated for a better understanding of the relationship between the composition and electrical properties. A cross-over from relaxor to ferroelectric state in BCTZ solid solution is evidenced similar to the one reported in ceramics. In KNLSTN single crystals, we observed a substantial evolution of the orthorhombic-to-tetragonal phase transition under minute composition changes.

## 1. Introduction

Piezoelectric materials may be used either to generate charges under stress (the direct effect) or to induce strain under an electric field (the reverse effect). Ferroelectric perovskite are of great importance among all the currently probed piezoelectric materials. The ideal cubic (C) structure has a general formula ABO_3_ and is described as a cubic unit cell with the corners occupied by a large cation (“A”, such as Pb, Ba, Ca, K, Na, *etc.*), the center by a smaller cation (“B”, such as Ti, Nb, Mg, Zr, *etc.*), and oxygen in the face’s center. The example of a complex perovskite with a great relevance to both fundamental and application-related issues is the standard piezoelectric material PbZr_1−*x*_Ti*_x_*O_3_ (PZT) [1]. An important amount of the research for environmentally-friendly ferroelectric materials aims at the replacement of lead-based ferroelectric and piezoelectric materials by equally good piezoelectrics, which contain no Pb. Outstanding piezoelectric constants have been obtained in (1−*x*)BaTi_0.8_Zr_0.2_O_3_-*x*Ba_0.7_Ca_0.3_TiO_3_ (BCTZ) [2] ceramics and in Li- and Ta-codoped (K,Na)NbO_3_ (KNLTN) [3]. These properties make these two lead-free systems promising as alternatives to lead-containing materials. Moreover single crystals should display better electromechanical properties so that it is expected that (K_0.5_Na_0.5_)NbO_3_-based (KNN) and BCTZ single crystals will exhibit much higher performances than the respective ceramics as previously predicted for BCTZ where a piezoelectric constant of about 1500–2000 pC·N^−1^ is anticipated [2].

In the present work, we focus on single crystal growth by top-seeded solution growth method of BCTZ and KNLTN substituted with antimony (KNLSTN). Single crystals of various compositions have been grown from high-temperature solutions. We focused our attention on BCTZ single crystals with compositions close to (Ba_0.850_Ca_0.150_)(Ti_0.900_Zr_0.100_)O_3_ (BCTZ50) for which the largest piezoelectric coefficients have been reported [2], and on Li_2_O-Na_2_O-K_2_O-Nb_2_O_5_-Ta_2_O_5_-Sb_2_O_3_ solid solutions in the vicinity of the morphotropic phase boundary (MPB). In addition to their very large expected piezoelectric efficiency, investigating these single crystals may be helpful to clear the way for understanding their ferroelectric/piezoelectric properties. Compared to previous investigations on ceramics, single crystals can lead to better chemical homogeneities, control, and improved understanding of the crystalline structure. As evidenced in the case of lead-containing piezoelectrics, this is a key step towards the improvement of the piezoelectric performances. For KNN crystals, based on previous investigations on ceramics, we chose KNN solid solutions doped with LiNbO_3_ where niobium was substituted by antimony and tantalum as referenced by Saito *et al.* [2] in order to reach the MPB composition where high and stable piezoelectric characteristics are expected. Hence, as referenced by Fu *et al.* [4] (Li*_x_*Na_0*.*52_K_0*.*48__−_*_x_*)(Nb_1__−_*_x_*_−_*_y_*Ta*_x_*Sb*_y_*)O_3_ composition with *x* = 0*.*04 and *y* = 0*.*06 is considered for its large piezoelectric coefficients (*d*_33_ = 335 pC·N^−1^) and high Curie temperature (*T*_c_ = 291 °C). Based on the few previous works related to the crystal growth from high-temperature solutions of BCTZ and KNLSTN [5,6], a common growth methodology of these solid solutions is presented for both these compounds, although there is no common ion between them and for which the phase diagrams remain unknown. Chemical analysis, both by electron probe microscopy analysis (EPMA) and inductively-coupled plasma-optical emission spectroscopy (ICP-OES) are displayed. The correlation between the crystalline symmetry, chemical content, and ferroelectricity is undertaken and the results are discussed in detail. Pyroelectric and piezoelectric measurements on some as-grown crystals are presented.

## 2. Experimental Procedure and Crystal Growth

### 2.1. Chemical and Physical Analysis

Single crystals exhibiting frequently growth twins and invariably ferroelectric domains with different sizes, room temperature X-ray diffraction experiments were performed on powders of crushed crystals using a PANalytical X’pert MPD diffractometer (PANalytical BV, Almelo, The Netherlands) with θ-θ Bragg Brentano configuration with a backscattering graphite monochromator for CuK_α1_ radiation (λ CuK_α1_ = 1.54056 Å). Full structural Rietveld and classical lattice parameter refinements have been performed on BCTZ and KNLSTN single crystals respectively. Laue back-scattering patterns were collected using a CCD-camera device (Photonic Science dual lens-coupled X-rays Laue system) after a 3–5 min stationary crystal irradiation with polychromatic X-rays supplied by a molybdenum anticathode. Single crystals were cut along pseudo-cubic (pc) directions with a diamond wire saw with an absolute accuracy less than 1°.

EPMA was achieved using a CAMECA SX-100 set-up (CAMECA SAS, Gennevilliers, France) with a wavelength dispersive spectrometer working at 15 kV in order to measure the concentrations of elements in the single crystals. EPMA reference samples (Table 1), single crystals or ceramics with 99.99% purity grade (4N), used for quantitative analysis of elements were chosen in order to reach a concentration accuracy of 1 mol %.

In addition, chemical analyses were done with a VARIAN 720-ES ICP-OES set up (Agilent Technologies Inc, Santa Clara, CA, USA) for lithium content. Samples weighing ~20 mg were dissolved in 10 mL mixture of HF (48%)-HNO_3_ (68%)-HCl (38%) with volume ratio 1:3:3, respectively, and 90 mL of distilled water. ICP-OES and EPMA analysis were performed in different regions of several samples from the same attempt and the given element concentration were averaged. The use of these two complementary chemical analyses is useful to fix the actual formula of crystals, a critical parameter for the optimization of ferroelectric/piezoelectric properties.

Relative concentration accuracy of elements by averaging EPMA and ICP-OES results is 0.24% for Li, 2.5% for Na and K, 0.5% for Nb, Ta, Sb, Ca and Ba, and 1% for Ti and Zr.

**Table 1:** Electron probe microscopy analysis (EPMA) reference samples for accurate quantitative analysis of elements.

Elements	EPMA References
Ba	BaTiO_3_ single crystal
Ca	CaF_2_ single crystal
Ti	BaTiO_3_ single crystal
Zr	ZrO_2_ sintered ceramics (4N)
Li	Undetected
Na	NaCl
K	KNbO_3_ single crystal
Nb	KNbO_3_ single crystal
Ta	Ta_2_O_5_ sintered ceramics (4N)
Sb	Metallic Sb polycrystal or Sb_2_O_3_ sintered ceramic (4N)

Prior to dielectric/piezoelectric investigations, the major faces of (001)_pc_ crystals were electroded using gold sputtering and silver wires attached to these electrodes with silver paste for dielectric experiments. The samples were set in a homemade cell enabling the temperature to be scanned from 80 up to 500 K. Prior to such a low-temperature run, the cell was pumped down and a slight overpressure of dry helium was introduced so as to avoid moisture adsorption. The samples were electrically connected to the output port of a HP4194 impedance analyzer (Hewlett Packard, Palo Alto, CA, USA) with an operating frequency range of 100 Hz–10 MHz. For piezoelectric and pyroelectric measurements, the samples were poled under a static electric field of about 8–10 kV·cm^−1^ during cooling from the Curie temperature. The piezoelectric constant *d*_31_ along with corresponding electromechanical coupling coefficient *k*_31_ for the length thickness extensional mode were determined at different temperatures by the resonance-antiresonance method on the basis of Institute of Electrical and Electronics Engineers (IEEE) standards [7]. A digital electrometer Keithley 6517B (Keithley Instruments Inc., Solon, OH, USA) was used to examine the temperature dependence of both the pyroelectric current and the spontaneous polarization *P*_s_.

### 2.2. Synthesis

Raw powders of BaCO_3_ (99.99%), CaCO_3_ (99.99%), TiO_2_ (99.99%), ZrO_2_ (99.99%) for BCTZ and of Na_2_CO_3_ (99.99%), Li_2_CO_3_ (99.99%), K_2_CO_3_ (99.99%), Nb_2_O_5_ (99.99%), Ta_2_O_5_ (99.99%), Sb_2_O_3_ (99.99%) for KNLSTN were weighed according to stoichiometric ratio of the selected compositions and their synthesis by solid state reaction are described by Benabdallah *et al.* and Prakasam *et al.* [5,6].

### 2.3. Growth Methodology

BCTZ and KNLSTN single crystals were grown by using the top-seeded solution growth method (TSSG). Growth attempts were carried out with an induction furnace in the case of BCTZ and with a two-zone resistive heating furnace for KNLSTN. BCTZ and KNLSTN solutions are considered, each, as a global solid solution including the solvent and the solute to be grown we assume that no defined compound or secondary parasitic phases could be obtained in the temperature range where the growth mechanisms occur. Hence, crystal growth attempts are carried out with a self-flux where an excess of TiO_2_ and an excess of Li_2_O and K_2_O could be considered as the solvent for BCTZ and KNLSTN growth, respectively.

In order to proceed to the first growth attempt of these solid solutions, the initial load composition, normalized with respect to stoichiometric ABO_3_ formula, is deduced from literature according to segregation coefficients which are either given or which can be calculated from phase diagram. In the case of no information in literature is available, the first growth load composition is targeted as that of the solute to be grown.

In the case of BCTZ, there are no data and no phase diagrams in the literature dealing with the BaO-CaO-TiO_2_-BaZrO_3_ pseudo-quaternary system from where the segregation coefficient of Ca, Ba, Ti and Zr could be obtained or calculated. Thus, the first solution composition was chosen according to the (Ba_0.850_Ca_0.150_)(Ti_0.900_Zr_0.100_)O_3_ formula (BCTZ50) owing to its colossal piezoelectric response found in ceramic sample. This composition is located near a phase convergence region [8] point of the phase diagram (Ba_0.7_Ca_0.3_TiO_3_)-(BaZr_0.2_Ti_0.8_O_3_) where a cubic non-ferroelectric phase can coexist with three ferroelectric phases of rhombohedral, orthorhombic and tetragonal symmetry.

In the case of KNLSTN, there are no data in literature dealing with phase relations in the pseudo-hexanary Li_2_O-Na_2_O-K_2_O-Nb_2_O_5_-Ta_2_O_5_-Sb_2_O_3_ system. We could have chosen Fu *et al.* [4] composition (with *x* = 0.04) as initial liquid composition, however, according to previous works [9,10,11,12,13,14,15,16,17,18,19,20,21,22,23,24,25,26,27], effective segregation coefficients of elements with respect to their molar fractions were either given or can be calculated and have shown a good reliability between them in ternary and quaternary systems containing Li, Na, K, Nb or Ta. Thus, the average segregation coefficients for the considered KNLSTN composition were chosen as follows: *k*_Li_ = 0.2 [12]; *k*_Na_ = 1.8 [11,20]; *k*_K_ = 0.25 [11]; *k*_Nb_ = 0.75 [17,22,24,27] and *k*_Ta_ = 3.5 [22,24,27] in order to reach the MPB. As no data dealing with Sb_2_O_3_- or Sb_2_O_5_-based phase diagram with constitutive compounds of KNLTN solid solution is available, antimony segregation coefficient was assumed to be equal to 2.

Having brought out the effective segregation of elements during the first growth attempt for which the liquid solution was corresponding to BCTZ50 and (Li_0.089_Na_0.128_K_0.783_)(Nb_0.967_Ta_0.009_Sb_0.024_)O_3_ compositions, the molar composition of the a next liquid composition were deduced from the polynomial regression fitted curves deduced from the chemical content of the previous attempts as a function of the liquid composition.

### 2.4. Crystal Growth

Following the optimization route described above, three and five crystal growth attempts were undertaken respectively for BCTZ and KNLSTN compositions.

At the present time, there are only few reports on the crystal growth of BaTiO_3_-CaTiO_3_ and BaTiO_3_-BaZrO_3_ solid solutions [28,29]. However, there is still no data in the literature dealing with the crystal growth process and the stabilization of the perovskite structure in BaTiO_3_-CaTiO_3_-BaZrO_3_ pseudo-ternary system except for BCTZ relaxor crystal growth carried out recently by Zeng *et al.* [30]. As no suitable seed with BCTZ composition was available, a 3-mm-thick iridium rod was used as a cold thermal point in order to initiate the nucleation and the growth of crystals. The rod was dipped into the melt after homogenization of the liquid solution for 3 h above the saturation temperature. A rotation speed ranging from 6 to 10 rpm was used with no pulling. The growing crystal mass was monitored during the whole process. The growth attempts took place on decreasing the temperature at a rate ranging between 0.5 and 1.5 °C·h^−1^. At the end of the growth process, the boules were set 5 mm above the liquid surface in order to reduce the thermal stress and cooled down to room temperature within 24 h.

For KNLSTN, the solution molten at a temperature above 1200 °C. The charge was held at a soaking temperature which is about 20 °C above the saturation temperature and stirred continuously with a platinum spatula at a rate of 40 rpm for 24 h under air atmosphere. As no suitable seed with KNLSTN composition was available, a platinum wire or spatula was used as the nucleation site for the crystal growth and a weighing device was used in order to measure the weight loss of the load in the crucible as well as the mass of growing crystals. The rotation speed was set between 8 and 20 rpm and decreasing thermal ramps ranging between 0.1 and 1 °C·h^−1^ were programmed. Measured longitudinal thermal gradient at the top of the liquid solution was around 1 °C·cm^−1^.

## 3. Results and Discussion

### 3.1. Morphology of Crystals

**Figure 1:**
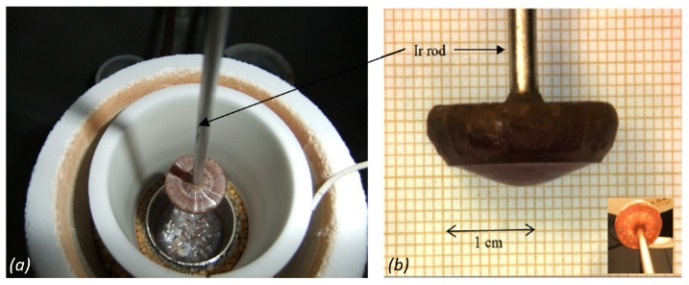
(**a**) (1−*x*)BaTi_0.8_Zr_0.2_O_3−_*x*Ba_0.7_Ca_0.3_TiO_3_ (BCTZ) as-grown boule in the inductively heated furnace with ceramics assembly for thermal insulation; and (**b**) as-grown centimeter sized BCTZ boule.

**Figure 2:**
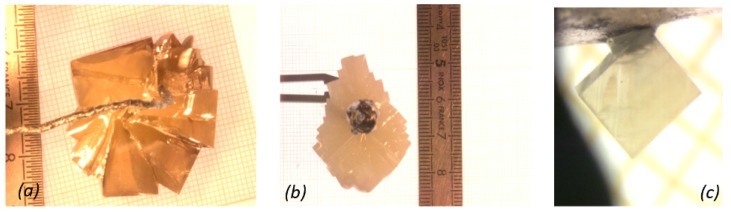
(**a**) Brownish-orange and (**b**) white-cloudy (Li*_x_*Na_0*.*52_K_0*.*48__−_*_x_*)(Nb_1__−_*_x_*_−_*_y_*Ta*_x_*Sb*_y_*)O_3_ (KNLSTN) centimeter-sized as-grown crystals obtained using top-seeded solution growth technique with different color; and (**c**) millimeter-sized as-grown single crystal on platinum wire exhibiting ferroelectric domains.

Millimeter to centimeter-sized crystals, around the iridium rod in the case of BCTZ (Figure 1) and agglomerated to the platinum wire for KNLSTN (Figure 2), with large regions devoid of flux, were obtained.

The BCTZ crystal growth attempts exhibited saturation temperatures of about 1485–1570 °C and yielded centimeter-sized boules delimited by radial cracks. These boules (Figure 1) displayed several single crystals dispersed all around the iridium rod with a cylindrical symmetry. Such obtained morphological features are assumed to be caused by the formation of spontaneous nucleation sites during the growth process around the iridium rod. Transparent and brownish single crystals free of cracks and twins were successfully extracted from the boules.

**Figure 3:**
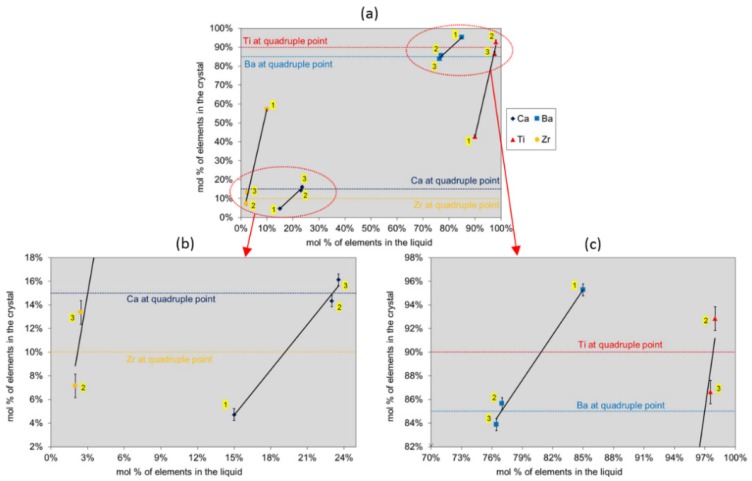
Segregation of elements in BCTZ for the three growth attempts (number of the attempt is written with a yellow background). (**a**) The molar concentrations of elements per site for BCTZ single crystals are plotted as a function of those corresponding to their initial liquid compositions. Dashed lines represent the Ca, Ba, Ti and Zr contents corresponding to BCTZ50 composition [2,8]; (**b**) Molar content of Ca and Zr as a function of their initial content in the solution; (**c**) Molar content of Ba and Ti as a function of their initial content in the solution.

**Figure 4:**
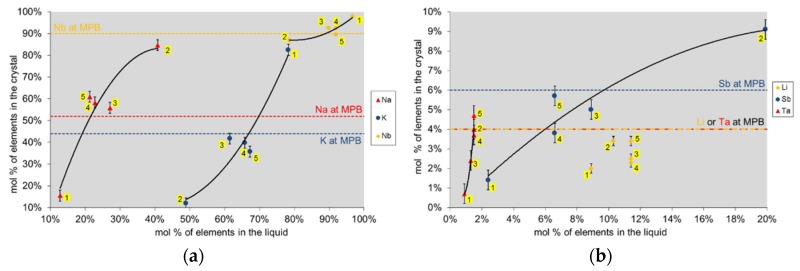
Segregation of elements in KNLSTN for the five growth attempts (number of attempt is written with yellow background). (**a**) Concentration of Na, K and Nb elements in crystal as a function of their initial content in liquid solution; and (**b**) concentration of Li, Sb and Ta elements in crystal as a function of their initial content in liquid solution. The dashed lines show the targeted compositions of Fu *et al.* [4].

In the case of KNLSTN, saturation temperatures ranged from 885 to 1200 °C. The crystals (Figure 2) exhibited clear region with a progressive brownish-orange color for the two first attempts. Crystals obtained in the last attempts show, with a random distribution, clear regions containing large domains with a 500-μm averaged size and white cloudy regions characterized by micro-sized domains of about a few tenths of micrometers. No chemical composition divergence between these two kinds of regions has been highlighted by EPMA for which the results of as-grown crystals composition analysis regarding the initial liquid solutions composition are given in Table 2 and Table 3 and depicted in Figure 3 and Figure 4.

In both cases, a substantial volatilization of the flux was observed. Volatilization rates of KNLSTN showed that the durations of growth with respect to the saturation temperature (Table 4) have an important role in the incorporation of alkali elements in A site and antimony in B site [6] whereas volatilization rate of BCTZ solution cannot be attributed to that. Indeed, continuous improvements of thermal insulation through ceramics assembly setup surrounding the crucible (Figure 1a) have been made for the second and third BCTZ growth attempt in order to decrease the high volatilization rate observed during the first BCTZ growth (Table 4). This lead to a substantial decreasing of the volatilization rates of BCTZ liquid solution whereas the duration was increasing from the first attempt to the third one. In general, we assume that TiO_2_ and alkali-based elements, especially Li- and Sb-based compounds are the main components of the volatilized powder for BCTZ and KNLSTN growths respectively.

**Table 2:** Normalized liquid and crystal compositions of (Li*_x_*Na_0*.*52_K_0*.*48__−_*_x_*)(Nb_1__−_*_x_*_−_*_y_*Ta*_x_*Sb*_y_*)O_3_ (KNLSTN) growth attempts.

KNLSTN Normalized Composition		Li (mol %)	Na (mol %)	K (mol %)	Nb (mol %)	Ta (mol %)	Sb (mol %)
No. Attempt-Name	Phase						
1st attempt KNL20S14T07N	Liquid solution	8.9%	12.8%	78.3%	96.7%	0.9%	2.4%
	Crystal	2.0%	15.5%	82.5%	97.9%	0.7%	1.4%
2nd attempt KNL34S91T40N	Liquid solution	10.3%	40.8%	48.9%	78.6%	1.5%	19.9%
	Crystal	3.4%	84.7%	11.9%	86.9%	4.0%	9.1%
3rd attempt KNL25S50T24N	Liquid solution	11.4%	27.1%	61.5%	89.8%	1.3%	8.9%
	Crystal	2.5%	55.8%	41.7%	92.6%	2.4%	5.0%
4th attempt KNL23S38T37N	Liquid solution	11.4%	22.8%	65.8%	91.9%	1.5%	6.6%
	Crystal	2.3%	58.3%	39.4%	92.5%	3.7%	3.8%
5th attempt KNL34S57T47N	Liquid solution	11.4%	21.3%	67.3%	91.9%	1.5%	6.6%
	Crystal	3.4%	60.9%	35.7%	89.6%	4.7%	5.7%
KNLSTN at morphotropic phase boundary (MPB)		4.0%	52.0%	44.0%	90.0%	4.0%	6.0%

**Table 3:** Normalized liquid and crystal compositions of (1−*x*)BaTi_0.8_Zr_0.2_O_3−_*x*Ba_0.7_Ca_0.3_TiO_3_ (BCTZ) growth attempts.

BCTZ Normalized Composition		Ca (mol %)	Ba (mol %)	Ti (mol %)	Zr (mol %)
No. Attempt-Name	Phase				
1st attempt BCTZ1	Liquid solution	15.00%	85.00%	90.00%	10.00%
	Crystal	4.72%	95.28%	42.72%	57.28%
2nd attempt BCTZ2	Liquid solution	23.00%	77.00%	98.00%	2.00%
	Crystal	14.35%	85.65%	92.84%	7.16%
3rd attempt BCTZ3	Liquid solution	23.54%	76.46%	97.55%	2.45%
	Crystal	16.22%	83.78%	85.38%	14.62%
BCTZ50		15.00%	85.00%	90.00%	10.00%

**Table 4:** Saturation temperatures, weight losses percentage, crystal growth durations, and weight loss rate due to volatilization with respect to the growth attempts for KNLSTN and BCTZ.

Growth Number-Name of the Attempt KNLSTN	*T*_saturation_ (°C)	Weight Loss (%)	Duration of Growth (h)	Weight Loss Rate (%·h^−1^)
1-KNL20S14T07N	885	3%	200	0.015
2-KNL34S91T40N	1195	5%	250	0.020
3-KNL25S50T24N	1190	<2%	100	<0.020
4-KNL23S38T37N	1200	7%	250	0.028
5-KNL34S57T47N	1200	<1%	50	<0.020
1-BCTZ1	1550–1570	~27%	120	0.225
2-BCTZ2	1500–1550	8%–9%	150	0.060
3-BCTZ3	1485–1510	6%–7%	300	0.023

We can infer that the first crystal growth attempt has shown a composition diverging significantly from expected MPB composition. Actually, in the case of KNLSTN, three elements are present per site involving a competition between ions as for their incorporation in the crystal lattice contrary to the assumption which took into account only two ions per site.

The effective segregation of Ti and alkali element is strongly dependent on the rate of volatilization of the liquid solutions of BCTZ and KNSLTN. As shown on EPMA and ICP-OES analysis graphs (Figure 3 and Figure 4), the lithium content cannot be fitted with a polynomial curve due to the high volatilization rate of Li for which the content in the five attempts is found to be randomly ranged from 2% to 3.4%. In the case of BCTZ, we note the substantial divergence of the Ti content in the third BCTZ composition than the second one. Expected Ti content about 90 mol % in the B site is not reached due to the reduction of volatilization induced by a rearrangement of ceramic assembly in the inductive furnace.

Hence, the control of stoichiometry of titanium, alkali elements, especially lithium, as well as antimony [6], is tricky due to the high saturation temperature, around 1500 and 1200 °C, respectively, for BCTZ and KNLSTN. Thermal insulation and stability of the growth furnace are the key-points for optimal single crystal growth of these lead-free crystals.

### 3.2. X-Ray Diffraction

**Figure 5:**
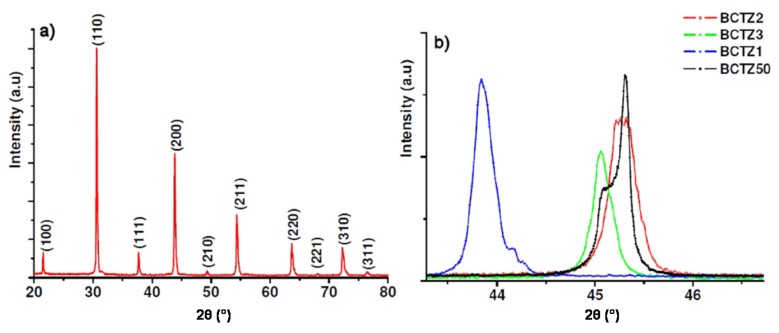
(**a**) X-ray powder diffraction pattern of the crushed as-grown BCTZ1 single crystal recorded at room temperature; and (**b**) shift of the position of the (200)_pc_ diffraction peak showing an increase of the unit cell volume in BCTZ1 compared to BCTZ50 composition. The lattice parameters were calculated using a Rietveld global profile-matching method. Although BCTZ2 and BCTZ3 could be indexed in the tetragonal symmetry a possible coexistence of different symmetries could not be excluded in these two compositions because of anisotropic line-shape broadenings along with no well-defined peak splitting.

Rietveld refinement and lattice parameters of BCTZ single crystals are given in Table 5. Figure 5a shows the X-rays powder diffraction (XRD) pattern of the as-grown BCTZ1 crystal (first crystal growth attempt) recorded at room temperature. The diffractogram indicates that BCTZ1 is of a cubic perovskite structure. Compared to the targeted BCTZ50 composition, all (hkl) diffraction peaks for BCTZ1 powder are shifted to lower 2θ angles, which correspond to an expansion of the unit cell. As the single crystal exhibits a slight variation of brownish color (Figure 1), the minor peak associated to (200) Bragg reflection in Figure 5b is assumed to be the contribution of a small amount of flux inclusions within the single crystal. As for BCTZ2 and BCTZ3, which refer to the second and third attempts, respectively, XRD analysis shows that the different chemical elements were successfully incorporated into A and B sites of the perovskite to form homogeneous solid solutions. Compared to BCTZ1, a decrease of the unit cell volume has been noticed for the latter compositions. It is revealed by the shift of (h00) Bragg peaks to higher angles. We can ascribe such a behavior to the decrease of concentrations of the elements with large ionic radius, like Zr and Ba, for the given solid solutions. This is consistent with the chemical contents reported above.

**Table 5:** Rietveld refinement for BCTZ crystals where a, b and c are lattice parameters and *R*_p_, *R*_wp_, *R*_exp_ and Χ^2^ are Rietveld reliability factors

Growth Number-Name of the Attempt BCTZ	Structure-Space Group	a (Å)	b (Å)	c (Å)	*R*_p_ (%)	*R*_wp_ (%)	*R*_exp_ (%)	Χ^2^
1-BCTZ1	Cubic-Pm-3m	4,123(1)	4,123(1)	4,123(1)	7.47%	9.96%	6.47%	2.37
2-BCTZ2	Tetragonal-P4mm	3,992(9)	3,992(9)	4,015(8)	7.67%	10.90%	8.51%	1.64
3-BCTZ3	Tetragonal-P4mm	4,010(3)	4,010(3)	4,021(4)	9.05%	12.40%	9.26%	1.80
BCTZ50	Tetragonal-P4mm	4,000(5)	4,000(5)	4,017(3)	6.88%	9.15%	6.29%	2.11

**Figure 6:**
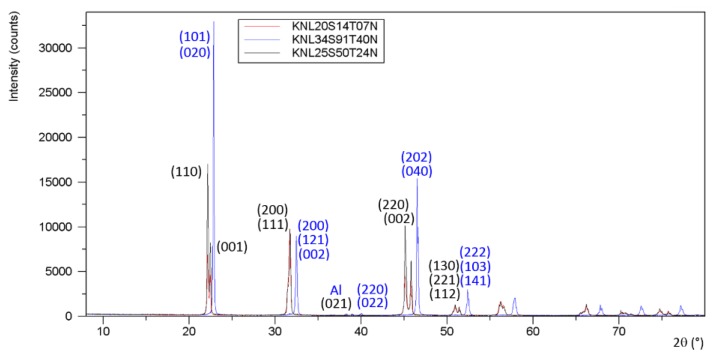
X-rays diffraction patterns on crushed crystals of KNL20S14T07N (attempt 1), KNL34S91T40N (attempt 2), and KNL25S50T24N (attempt 3) exhibiting an orthorhombic structure.

**Figure 7:**
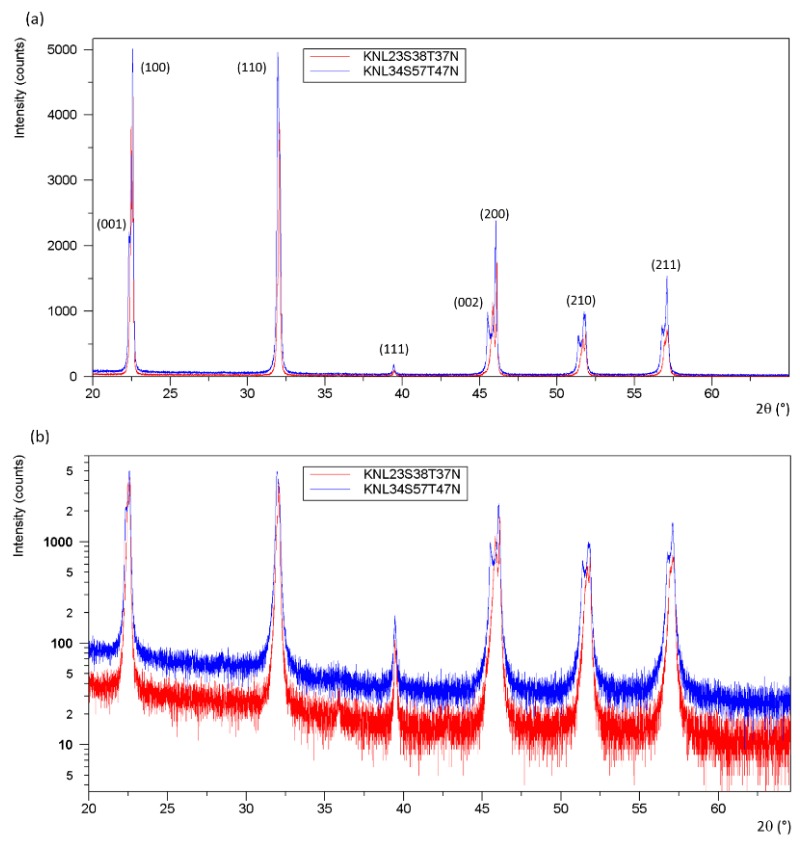

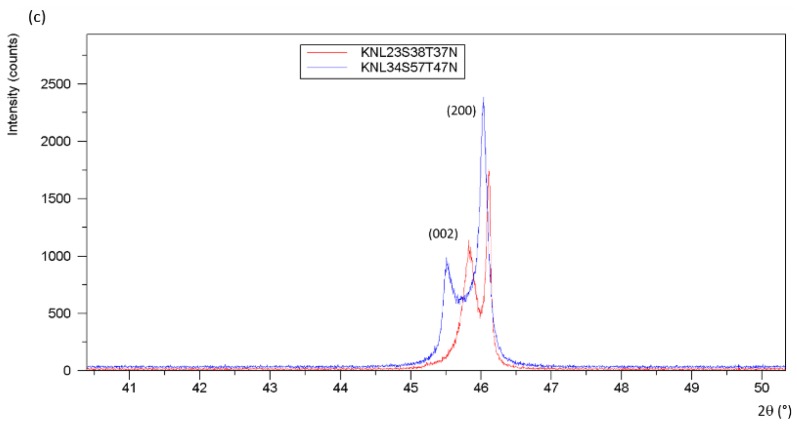
XRD patterns on crushed crystals of (**a**) KNL23S38T37N (attempt 4) and KNL34S57T47N (attempt 5) exhibiting tetragonal structure; (**b**) XRD patterns with an intensity logarithm scale where no inclusions are detected (**c**) and zoom on (200) and (002) lines for KNL23S38T37N and KNL34S57T47N.

The powder XRD patterns of crushed single crystals corresponding to KNL20S14T07N (attempt 1), KNL34S91T40N (attempt 2), and KNL25S50T24N (attempt 3) revealed orthorhombic perovskite structures with KNbO_3_-type-like cells for KNL20S14T07N and KNL25S50T24N, and NaNbO_3_-type-like cells for KNL34S91T40N (Figure 6). Concerning KNL23S38T37N (attempt 4) and KNL34S57T47N (attempt 5), Figure 7a exhibits diffraction peaks corresponding to the perovskite phase. No presence of inclusions is detected in the background of the logarithmic XRD pattern of these materials (Figure 7b). XRD patterns of KNL23S38T37N and KNL34S57T47N are consistent with tetragonal state but the shifting of the (002) line towards low angles is highly sensitive to little change of compositions (Figure 7c). Following Fu *et al.* [4], we note that samples KNL23S38T37N and KNL34S57T47N are close to the switching between orthorhombic and tetragonal symmetry at room temperature.

### 3.3. Dielectric Measurements on BCTZ and KNLSTN Crystals

#### 3.3.1. BCTZ Characterization

**Figure 8:**
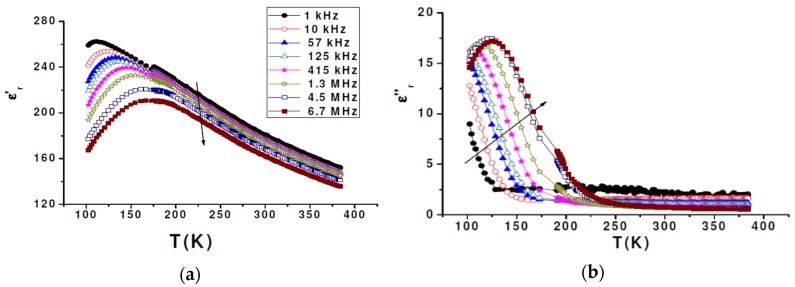
Real (**a**) and imaginary (**b**) parts of the complex dielectric permittivity, ε_r_’ and ε_r_’’, respectively, as a function of temperature and frequency for BCTZ1 (arrows indicate the direction of increasing frequency).

The temperature dependence of the real ε_r_’ and imaginary ε_r_’’ parts of the complex dielectric permittivity (ε = ε_r_’ + *j* × ε_r_’’) of BCTZ1 are displayed in Figure 8, showing the whole frequency range between 1 kHz and 1 MHz. Both these parameters prove the usual trends of relaxor. ε_r_’ and ε_r_” are frequency-independent at high temperature and ε_r_’ increases on cooling. Moreover, ε’_r_ undergoes a broad peak whose corresponding temperature *T*_m_ increases, while the amplitude ε_rmax_’ decreases with increasing frequency. At temperatures lower than this maximum, ε_r_’ curves are dispersed and parallel. The imaginary part of the relative dielectric constant displays frequency dependent maxima whose amplitude increases with the operating frequency, *i.e.*, opposite to the real part. All these qualitative features are in perfect agreement with the archetype relaxor compound Pb(Mg_1/3_Nb_2/3_)O_3_ (PMN) [31,32]. Referring to the systematic investigation of the BaTiO_3_-CaTiO_3_-BaZrO_3_ phase diagram of powders and ceramics [33], we find a good agreement between the Zr and Ba segregation in BCTZ single crystals and their relaxor features. Indeed, when the Zr content at B-sites of the perovskite exceeds 27 mol %, a shift from ferroelectric to relaxor state is observed. Thus, starting from the initial ferroelectric composition with Zr content = 15 mol %, we obtained a relaxor with Zr content of 57.3% In this work, we think that the substitution of barium (Ba^2+^) by calcium (Ca^2+^) stabilizes more and more the perovskite structure but it does not alter significantly functional properties of BCTZ solid solutions: relaxor characteristic signatures are solely governed by the Zr content in B-sites.

**Figure 9:**
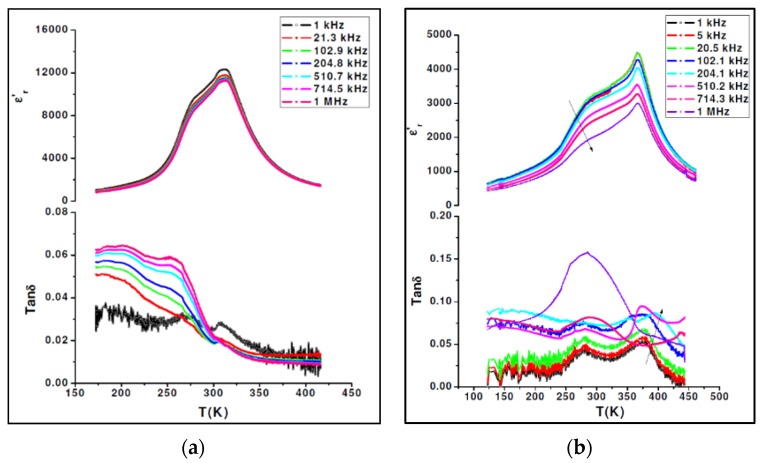
(**a**) Dielectric constant and loss tangent as a function of temperature and frequency of a randomly oriented BCTZ2 sample; and (**b**) temperature dependence of dielectric constant and loss tangent at various frequencies of [001]_pc_ oriented BCTZ3 single crystal (arrows indicate the direction of increasing frequency).

The dielectric behavior of the randomly oriented BCTZ2 sample of composition (Ba_0.857_Ca_0.143_)(Ti_0.928_Zr_0.072_)O_3_, which contains a much higher Ti content, displays (Figure 9a) dielectric constant and loss with a broad frequency-independent anomaly at 310 and 275 K. These two peaks are the signature of two successive phase transitions, which are in full agreement with what was reported in ceramics of similar composition. Figure 9b shows the temperature dependence of the dielectric constant and loss at various frequencies for (001)_pc_-oriented BCTZ3 single crystal. Dielectric parameters confirmed the chemical analysis displaying well defined anomalies at 366 and 265 K. The intermediate phase was thus much more extended than that depicted in BCTZ2, which was already ferroelectric.

Another feature, which was not observed in ceramics, is the very peculiar frequency behavior of the dielectric permittivity. While it is almost dispersion-less in the high-temperature cubic phase and in the low temperature phases, it shows a strong dispersion in the vicinity of dielectric anomalies. Even though the temperature at which the anomalies occur is not frequency dependent, a strong depletion of the permittivity is observed when the frequency increases. Such phenomena could be attributed to ferroelectric domain wall motions, which translate into an increasing of the dielectric loss with increasing frequency. In BCTZ solid solutions, we naturally expect compositional fluctuations and/or structural disordering of cations in one or more crystallographic sites of the perovskite structure. These microscopic characteristics will systematically disturb the long-range dipolar interactions and induce a diffuse behavior within the ferroelectric phase. Moreover, it cannot be excluded that this feature could be attributed to phase transitions as previously seen in literature [2,8,34].

**Figure 10:**
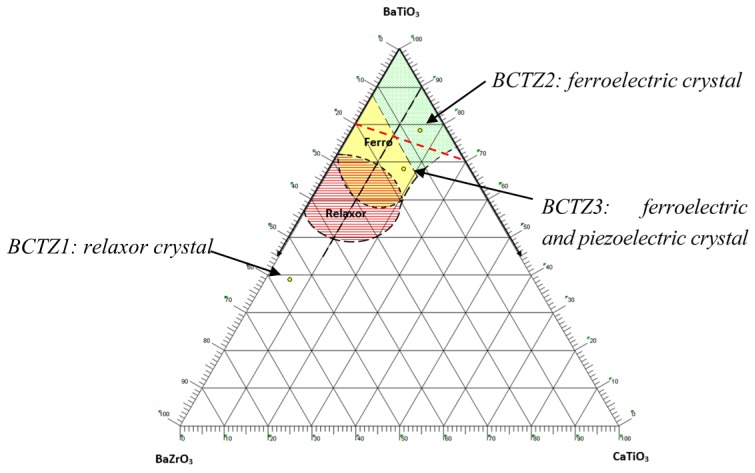
Molar pseudo-ternary phase diagram BaTiO_3_-BaZrO_3_-CaTiO_3_ three different crystals compositions (yellow discs) are inserted in the relaxor-ferroelectric zones described by Ravez *et al.* [33]. Dielectric behaviors of the crystals are in accordance with ceramics of same compositions [5,33]. Red dashed line corresponds to the (1−*x*)BaTi_0.8_Zr_0.2_O_3_-*x*Ba_0.7_Ca_0.3_TiO_3_ promising piezoelectric compositions above mentioned.

As a general result, dielectric behaviors of the three BCTZ crystals exhibit the same trend than that described by Ravez *et al.* [33] with ceramics of same compositions (Figure 10).

**Figure 11:**
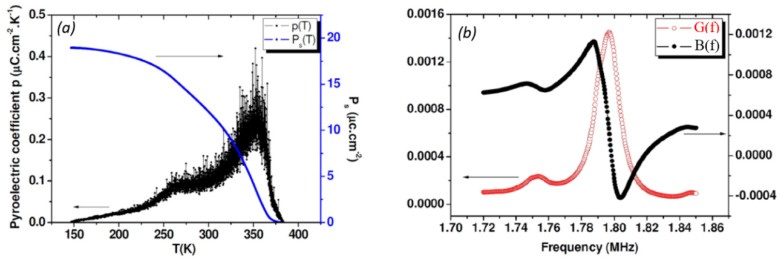
(**a**) Temperature dependence of the pyroelectric coefficient *p* and spontaneous polarization *P*_s_ of the [001]_pc_ oriented BCTZ3 single crystal; and (**b**) resonance curves for the length thickness extensional mode measured at 337K for [001]_pc_ oriented BCTZ3 single crystal. The plot shows a frequency dependent sweep of the conductance G and the susceptance B, which are the real and imaginary components of the admittance, respectively.

Pyroelectric and piezoelectric measurements in BCTZ3 are depicted in Figure 11 which illustrates the temperature dependence of the pyroelectric coefficient p along with the spontaneous polarization *P*_s_. Two anomalies are detected corresponding to cubic-tetragonal-rhombohedral phase transitions. Contrary to what has already been observed in BCTZ ceramic samples, *P*_s_ shows less sensitivity to the temperature change in BCTZ3 single crystal. The maximum polarization remains; however, below 20 μC·cm^−2^ (Figure 11a). The piezoelectric response for the length thickness extensional mode is determined at different temperatures through frequency-dependent sweeps of the conductance G and the susceptance B (Figure 11b).

Table 6 summarizes results obtained for three selected temperatures. It can be seen that the electromechanical response of BCTZ3 is smaller than that observed in BCTZ50 ceramic sample close to room temperature (*d*_31_ (BCTZ50 ceramic disk) > *d*_31_ (BCTZ3 single crystal plate) near room temperature).

**Table 6:** Piezoelectric constants d_31_ and electromechanical coupling factors *k*_31_ calculated at three selected temperatures for BCTZ3 crystal elongated bar oriented along pseudo-cubic [001]_pc_ direction.

*T*(K)	*d*_31_ (pC·N^−^^1^)	*K*_31_
293	70	0.14
305	93	0.18
337	91	0.17

To achieve large piezoelectric coefficients, we applied poling field of about 5 kV/cm which is larger than the coercive field which is 2 kV·cm^−1^ at most [5]. The polarization of 20 μC·cm^−2^ (Figure 11a) confirmed that a saturated state was reached. However, it can be seen that the electromechanical response of BCTZ3 is smaller than that observed in BCTZ50 ceramic sample close to room temperature. Some possibilities can be suggested to understand such a difference:

- Common resonance geometries along with recommended aspect ratios are usually used to characterize piezoelectric materials. In our case, the dimensions of BCTZ3 single crystal do not follow appropriately the metric conditions for length thickness extensional mode. Therefore, we may introduce a significant error when determining the electromechanical coefficients.
- The ferroelectric and piezoelectric responses possess anisotropic behaviors in single crystals and optimized crystallographic orientation is required to increase the piezoelectric efficiency [35,36,37].
- Although the BCTZ3 composition is close to the targeted BCTZ50 from the chemical point of view, both of them behave differently from the crystallographic point of view. Based on recent studies of Keeble *et al.* [8]; the orthorhombic O-phase found in BCTZ50 close to ambient temperature seems to be absent in BCTZ3 (from dielectric and pyroelectric measurements). Consequently, BCTZ3 is far away from the phase convergence region already reported for BTZ-BCT pseudo-binary phase diagram and no instability gradient could then be attained. This situation may explain the relatively low piezoelectric response in BCTZ3 compared to BCTZ50 ceramics composition. Finally, it is perhaps interesting to state that electromechanical properties of BCTZ3 single crystal presented, in this work, are consistent with those reported for BTZ (BaTiO_3_-BaZrO_3_) and BCT (BaTiO_3_-CaTiO_3_) single crystals synthesized by laser heated pedestal growth and floating zone techniques, respectively [29,38].

#### 3.3.2. KNLSTN Characterization

**Figure 12:**
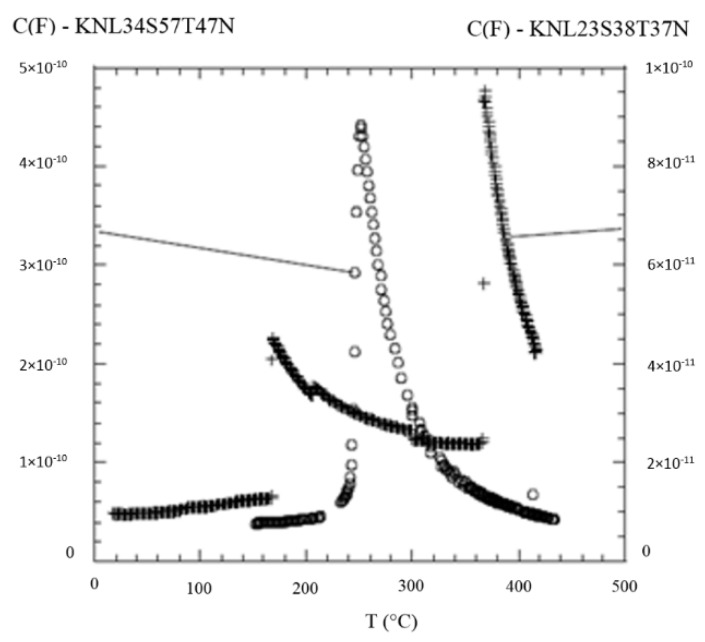
Variation of dielectric properties with temperature for KNL23S38T37N and KNL34S57T47N. (Reproduced from [6]. Copyright © 2012 Elsevier Masson SAS. All rights reserved).

The dielectric measurements carried out on all KNLSTN samples displayed no dielectric anomaly on the samples one to three from room temperature up to 420 °C. The results of KNL23S38T37N and KNL34S57T47N exhibit ferroelectric properties. The temperature dependence of the dielectric constant is shown in Figure 12. While two peaks are observed at 370 and 160 °C for KNL23S38T37N, only one single ferroelectric transition was recorded on KNL34S57T47N at 250 °C. All these anomalies can be ascribed to ferroelectric transitions because they are frequency independent. In KNL34S57T47N, the extrapolated Curie temperature was found to be 248 °C, close to the temperature of the dielectric maximum of 250 °C. This confirms that the ferroelectric transition is of a second order type. KNLSTN ceramics of composition close to our single crystals exhibit two phase transition temperatures, an orthorhombic (O) to tetragonal (T) transition temperature *T*_(O–T)_ at around 200 °C and a tetragonal to cubic (C) transition temperature *T*_(T–C)_ at 400 °C [38]. These are close to our reported transition temperatures in KNL23S38T37N where *T*_(O–T)_ = 160 °C and *T*_(T–C)_ = 370 °C and for which a deficit of lithium has been observed. We, thus, confirm that KNLSTN single crystals have the same succession of phase transition than ceramics of the same composition.

We next compare the ferroelectric transition temperature of KNL23S38T37N and KNL34S57T47N. We see a down-shift of the first transition temperature *T*_(T–C)_ of more than 100 °C in KNL34S57T47N. From the chemical analysis reported above, the main trend is that KNL34S57T47N includes more Li than KNL23S38T37N (3.4 mol % instead of 2.3 mol %). Such strong depression of ferroelectric transition temperature upon increasing Li substitution was also reported by Jimenez *et al.* [39] in ceramics where larger amount of Li in the starting reagents was needed in order to get such a large transition temperature shift. In addition, Hollenstein *et al.* [40] and Ochoa *et al.* [41] found that the addition of Li in the A site and Ta, Sb in the B site in ceramics decrease this temperature. As we discussed, from the powder XRD patterns of KNL23S38T37N and KNL34S57T47N, for which ferroelectric measurements were made, we noticed that KNL23S38T37N and KNL34S57T47N are close to the switching between orthorhombic and tetragonal symmetry at room temperature and, as a consequence, the ferroelectric behaviors of KNL23S38T37N and KNL34S57T47N are drastically different, as can be seen from the sizable differences in transition temperatures (Figure 12).

The large change of ferroelectric transition temperature with minute change of composition is similar to what is observed in the MPB region of lead containing materials like PZT [31] or lead-free BCTZ [2] solid solutions. This similarity is an indication for possible large piezoelectric coefficients of KNLSTN single crystals, but this still remains an open question.

Piezoelectric constant for KNL34S57T47N was measured by the Berlincourt method and gave, at room temperature, around *d*_33_ = 39 pC·N^−1^ on a randomly-orientated crystal. This is a smaller value than that expected and mentioned by Fu *et al.* [4], which, as well as the Curie temperature, could be attributed to the elements stoichiometry differences between our composition and theirs as well as slight chemical inhomogeneities in the bulk crystal. Moreover, the low value of this direct piezoelectric effect measurement could be due to inhomogeneity of the applied stress caused by the non-optimal shape of the contact between the sensor and the sample.

## 4. Conclusions

In this paper, we presented the results of BCTZ and KNLSTN single crystals which have been successfully grown by the flux method. Our growth methodology for these both kinds of materials consisted in considering the whole solution composition including the solvent and the solute to grow. The solvents acting as self-flux were chosen as TiO_2_ for BCTZ solution and, mainly, Li_2_O and K_2_O for KNLSTN. Centimeter-sized boule grown without seeds was obtained. Necessary and sufficient millimeter-oriented samples could have been extracted and shaped in order to perform suitable chemical and physical analysis.

EPMA and ICP-OES analysis of BCTZ and KNLSTN crystals revealed that the as-grown crystals are enriched with zirconium and barium, and tantalum and sodium, respectively, due to the difference of segregation of cations in the solid solution and the volatilization of titanium-based and lithium-based compounds, respectively.

In the case of BCTZ crystals our first two attempts showed that a continuous cross-over from relaxor to the ferroelectric state can be achieved in BCTZ lead-free materials. This result could be considered as a first step toward further optimization of the piezoelectric properties of BCTZ single crystals. Based on three growth trials, we were able to tune the starting composition to reach (Ba_0.838_Ca_0.162_)(Ti_0.854_Zr_0.146_)O_3_ composition (BCTZ3) which is very close to the best piezoelectric BCTZ50 composition. This single crystal seems to display a very interesting coexistence between ferroelectric and relaxor states, which led to a reasonably high transverse piezoelectric coefficient *d*_31_ = 93 pC·N^−1^ near room temperature.

Characterization of KNLSTN crystals highlighted a huge loss of the alkali compounds, particularly lithium with respect to the duration of growth. Two KNLSTN single crystals with close compositions have shown two types of sharp different dielectric features which have been correlated to the change of structure. Room temperature XRD analysis identified the presence of pure perovskite phase with tetragonal symmetry in both samples, the first sample (KNL23S38T37N) exhibited ferroelectric responses like orthorhombic structure and characterized by a dielectric transitions at around *T*_(O–T)_ = 160 °C and then a ferroelectric to paraelectric one measured at *T*_(T–C)_ = 370 °C. The second sample (KNL34S57T47N) displayed one ferroelectric to paraelectric transition at *T*_(T–C)_ = 248 °C.

Further growth attempts of BCTZ and KNLSTN will enable us to increase the size and the homogeneity of the single crystals and for obtaining better piezoelectric properties which are of interest not only for lead-free but also for classical lead-containing piezoelectrics.
